# Adverse outcomes post-COVID-19 hospitalization among ESRD patients: A retrospective cohort study in 5 California university medical centers

**DOI:** 10.1371/journal.pone.0332203

**Published:** 2025-09-18

**Authors:** Marc Reiterman, Andrew I. Chin, Heejung Bang

**Affiliations:** 1 Graduate Group in Epidemiology, University of California, Davis, California, United States of America; 2 Department of Internal Medicine, School of Medicine, University of California, Davis, California, United States of America; 3 Division of Biostatistics, Department of Public Health Sciences, University of California, Davis, California, United States of America; Warren Alpert Medical School of Brown University: Brown University Warren Alpert Medical School, UNITED STATES OF AMERICA

## Abstract

**Background:**

Individuals with end-stage renal disease (ESRD) hospitalized for COVID-19 may have a higher risk for adverse post discharge events. To investigate these risks, we examined a large database of individuals admitted for COVID-19 and discharged alive, to see if ESRD was independently associated with higher risk for death within 180 days or for 30-day hospital readmission.

**Methods:**

We retrospectively compared risks for hospital, non-hospital, and overall death within 180 days and 30-day hospital readmission between individuals with and without ESRD. We studied individuals hospitalized with COVID-19 between January 6, 2020 and August 3, 2023, at any of the 5 University of California medical centers and discharged alive.

**Results:**

Of 11,406 individuals hospitalized with COVID-19 who were discharged alive, 713 (6.3%) had ESRD. Compared to individuals without ESRD, those with ESRD had a significantly higher hazard of hospital and overall death within 180 days post-discharge in the unadjusted analyses, but not in the analyses adjusted for demographic variables, hospitalization period, and comorbid conditions (adjusted Wald chi-square hazard ratio (HR) 1.36, 95% Wald CI 0.78–2.37; adjusted HR 1.05, 95% CI 0.73–1.51, respectively). Those with ESRD did not have a significantly higher hazard of non-hospital death within 180 days post-discharge in either the unadjusted or the adjusted analyses (adjusted HR 0.89, 95% CI 0.55–1.44), but did have a significantly higher hazard of 30-day hospital readmission in both the unadjusted and the adjusted analyses (adjusted HR 1.36, 95% CI 1.14–1.63, p = 0.001).

**Conclusions:**

ESRD patients hospitalized with COVID-19 had a higher unadjusted risk of hospital and overall death within 180 days and of 30-day hospital readmission than individuals without ESRD. After adjusting for demographic factors, hospitalization period, and comorbidities, presence of ESRD was not found to significantly increase the risk for hospital, non-hospital, or overall death within 180 days post-discharge, but was found to significantly increase the risk of 30-day hospital readmission.

## Introduction

Since December 2019, infection with SARS-CoV-2, the virus that causes COVID-19, has led to one of the world’s most serious infectious disease pandemics. While most individuals affected by SARS-CoV-2 exhibit only mild symptoms, many individuals require hospitalization, with the most acute patients needing intensive care unit (ICU) admission, mechanical ventilation and extracorporeal membrane oxygenation (ECMO) treatment [1]. Infection is the second most reported cause of death for those on dialysis [2], with annual death rates from pneumonia and sepsis substantially higher than that of the general population [3]. ESRD patients have a compromised and poorly regulated immune system, which may increase susceptibility to bacterial and viral infections, including the SARS-CoV-2 virus [4].

In a retrospective study of 1,344 adult COVID-19 infected patients discharged alive from the emergency department or hospital between March 3 and May 15, 2020 in a large New York City health system, Kingery et al. [5] examined the risks for rehospitalization within 30 days and overall (combined hospital and non-hospital) death within 30 days post-discharge among individuals post-discharge for COVID-19 infection with ESRD (N = 75) compared to those without. After adjusting for numerous demographic and comorbid conditions, including “socio-demographics (age, sex, race, housing status), length of stay, comorbidities (tobacco use, body mass index [BMI], cardiovascular disease, chronic kidney disease, diabetes, chronic lung disease, HIV, active cancer, and other immunocompromised state), symptoms (fever, cough, diarrhea, nausea/vomiting, myalgia, dyspnea), and chest imaging at presentation; clinical course (new-onset myocardial infarction/arrhythmia/heart failure, dialysis, vasopressor requirement, hypoxia upon presentation, intubation/extubation, and tracheostomy) and infection parameters (PCR viral load, respiratory viral pathogen panel, and blood culture)”, the authors found a significantly higher hazard of rehospitalization within 30 days (adjusted hazard ratio (HR) 2.94, 95% CI 1.78–4.84, p < .0001) among individuals post-discharge with ESRD compared to those without, but no difference in the hazard of overall death within 30 days post-discharge for these two groups (adjusted HR 1.22, 95% CI 0.29–5.12, p = 0.789). In a larger national cohort study by Verna et al [6] of 29,659 patients hospitalized with COVID-19 at one of 297 hospitals across 40 US states between February 15 and June 9, 2020, patients with ESRD (N = 1,336) were also found to have a significantly higher odds of 30-day hospital readmission (adjusted odds ratio (OR) 2.27, 95% CI 1.81–2.86) and a higher, but not statistically significant odds of death during hospital readmission (adjusted OR 1.43, 95% CI 0.63–3.24). Covariates adjusted for in their statistical model included age, sex, length of index hospital stay, census region, insurance type, and the comorbidities chronic kidney disease, ESRD, collagen vascular disease (CVD), hypertension, obesity, cerebrovascular disease, pulmonary disease, diabetes, smoking, cardiac disease, respiratory disease, and sepsis/systemic inflammatory response syndrome. Interestingly, this study also found a difference in the risk of 30-day readmission by geographical area, with odds higher in the United States Northeast compared with either the West or the South.

Huang et al. [7] conducted a retrospective cohort study of 2,180 patients discharged from 15 Kaiser Permanente Southern California medical centers between April 1 and July 31, 2020. After adjusting for age, sex, race, BMI, Charlson Comorbidity Index (a tool used to predict long-term mortality in patients with multiple comorbidities), days from diagnosis to index hospital admission, mechanical ventilation, oxygen therapy, length of stay and discharge disposition for index hospitalization, and the comorbidities chronic pulmonary disease, diabetes, hypertension kidney disease, and chronic heart failure, the authors found that patients with kidney disease had a higher, but not statistically significant, odds of rehospitalization (adjusted OR 1.35, 95% CI 0.81–2.26, p = 0.25). A large meta-analysis of 28 national and international studies of 68,236 patients hospitalized for COVID-19 conducted by Akbari et al [8] found that patients with kidney disease had a significantly higher hazard of rehospitalization within 30 days (adjusted OR 2.52, 95% CI 1.23–2.85, p < .0001). Lastly, in a study of 222,154 Medicare beneficiaries on dialysis with 436,745 live acute-care hospital discharges from 7871 Medicare-certified dialysis facilities between January 1 and October 31, 2020, Wu et al [9] found a significantly higher hazard of rehospitalization (adjusted HR 1.59, 95% CI 1.53–1.65, p < 0.001) for patients with a diagnosis of COVID-19.

It has been documented that ESRD patients in the United States had a high unplanned hospital 30-day readmission rate prior to the COVID-19 pandemic. The United States Renal Data System Annual Data Report lists national year 2020 30-day rehospitalization rates of 39.8% for ESRD patients on hemodialysis and 38.5% for ESRD patients on peritoneal dialysis. A study by Donnelly et al [10] of patients admitted with COVID-19 to the Veterans Affairs Healthcare System found that risk for adverse clinical outcomes following a hospitalization for COVID-19 is highest in the 7–10 days following discharge. For some diseases such as heart failure, hospital readmission rates during the pandemic may have been lower than historical norms due to the impacted hospital system and the desire to reduce patient exposure to other hospitalized COVID-19 patients.

In our study, we examined the post-hospital discharge outcomes of ESRD patients admitted for COVID-19 in a diverse Western United States population. We compared outcomes post-hospitalization for COVID-19 in ESRD patients to that of individuals without ESRD, at 5 University of California academic medical centers. Our objective was to study if ESRD patients hospitalized with COVID-19 had a significantly higher adjusted risk of hospital, non-hospital, or overall death within 180 days or of 30-day hospital readmission, compared with those who did not have this disease.

## Methods

We used the University of California COVID Research Database (UC CORDS) which is a large, harmonized database provided by the UC Health Data Warehouse that includes patients from the 5 tertiary care, academic hospitals of the University of California (located in Davis, Irvine, Los Angeles, San Diego and San Francisco). Written informed consent was not required to conduct this study. It was granted an exemption for human subjects protection by the UC Davis Institutional Review Board (protocol# 1604619–1).The authors had access to information that could potentially identify individual participants during or after data collection (the data were accessed for research purposes on 12/22/2023), since the detailed pseudonymized patient data used for this study were potentially re-identifiable. This retrospective longitudinal study included adults with and without ESRD, who were 18 years or older when first hospitalized for COVID-19 (qualifying or index hospital admission), and who tested positive for COVID-19 by polymerase chain reaction nasal swab within 30 days prior to or at any time during their hospital stay. We included all adult patients with COVID-19 admitted between January 6, 2020 and August 3, 2023, and discharged alive by August 4, 2023, with 3 exceptions: individuals were excluded from the study if, for their index hospital admission, they were transferred into the University of California hospital system from a hospital outside this system (N = 1,053), they were transferred out of the University of California hospital system (N = 503), or they were admitted to an inpatient obstetric service (N = 259). The study end date was August 4, 2023. Individuals were classified as having ESRD if their index hospital admission included the International Classification of Diseases, 10^th^ revision, Clinical Modification (ICD-10-CM) code N18.6. We excluded individuals with an active renal transplant from the ESRD group but included individuals with failed kidney transplants who were back on long-term dialysis.

Demographic and baseline characteristics included age in years at the index hospital admission, sex, race and ethnicity. Self-reported data on race and ethnicity were combined and categorized as White, Hispanic, Black, Asian, and Other/Unknown. The date of the index hospital admission was grouped into hospitalization periods for each of the COVID-19 waves which occurred during the study period. Comorbid conditions identified in individuals with/without ESRD prior to or on the date of their qualifying hospital admission are listed in Table 1. Individuals were classified as having or not having these comorbid conditions by utilizing ICD-10-CM codes.

**Table 1:** Demographic and clinical characteristics of individuals with/without ESRD Post-Hospital Discharge for COVID-19 (N = 11,406).

Variable		Non-ESRD (N = 10,693)	ESRD (N = 713)
Age (years), median, (IQR)		60 (44,74)	61 (49,72)
Age (%)	18–39	2084 (19.5)	102 (14.3)
	40–49	1275 (11.9)	78 (10.9)
	50–59	1828 (17.1)	140 (19.6)
	60–69	2079 (19.4)	189 (26.5)
	70–79	1659 (15.5)	121 (17.0)
	80+	1768 (16.5)	83 (11.6)
Sex	Male	5810 (54.3)	400 (56.1)
Race/Ethnicity	Asian American	1295 (12.1)	80 (11.2)
	Black	853 (8.0)	86 (12.1)
	Hispanic	3921 (36.7)	349 (48.9)
	Other/Unknown	1321 (12.4)	72 (10.1)
	White	3303 (30.9)	126 (17.7)
Index Hospitalization Period	Jan-May 2020	356 (3.3)	24 (3.4)
	Jun-Aug 2020	993 (9.3)	68 (9.5)
	Sep 2020-Jan 2021	2894 (27.1)	200 (28.1)
	Feb-May 2021	551 (5.2)	27 (3.8)
	Jun-Nov 2021	925 (8.7)	34 (4.8)
	Dec 2021-Feb 2022	1537 (14.4)	141 (19.8)
	Mar-Sep 2022	1587 (14.8)	91 (12.8)
	Oct 2022-Aug 2023	1850 (17.3)	128 (18)
COVID-19 Vaccinations Prior to Index Hospitalization	Missing	8560 (80.1)	581 (81.5)
	1	227 (2.1)	5 (0.7)
	2	644 (6.0)	44 (6.2)
	3	635 (5.9)	40 (5.6)
	4	298 (2.8)	18 (2.5)
	5+	329 (3.1)	25 (3.5)
ADI^1^	Missing	1087 (10.2)	38 (5.3)
	1	1371 (12.8)	55 (7.7)
	2	981 (9.2)	52 (7.3)
	3	998 (9.3)	57 (8.0)
	4	1158 (10.8)	76 (10.7)
	5	1165 (10.9)	82 (11.5)
	6	1084 (10.1)	107 (15)
	7	949 (8.9)	86 (12.1)
	8	772 (7.2)	72 (10.1)
	9	602 (5.6)	42 (5.9)
	10	526 (4.9)	46 (6.5)
**Comorbid Condition**			
Major Cardiac Disease		5837 (54.6)	651 (91.3)
Valvular Cardiac Disease		1410 (13.2)	204 (28.6)
Cardiac Arrhythmias		4200 (39.3)	402 (56.4)
Peripheral Vascular Disease		940 (8.8)	203 (28.5)
Coagulopathy		2463 (23.0)	317 (44.5)
Cancer		2180 (20.4)	108 (15.1)
Diabetes		3809 (35.6)	491 (68.9)
HIV/AIDS		139 (1.3)	13 (1.8)
Hypertension		6436 (60.2)	690 (96.8)
Hyperthyroidism		1585 (14.8)	169 (23.7)
Liver Disease		1804 (16.9)	189 (26.5)
Chronic Neurological Conditions		2042 (19.1)	163 (22.9)
Obesity		2997 (28.0)	272 (38.1)
Paralysis		480 (4.5)	37 (5.2)
Chronic Psychoses		444 (4.2)	24 (3.4)
Pulmonary Disease		3263 (30.5)	295 (41.4)
Rheumatoid Arthritis/CVD		729 (6.8)	74 (10.4)
Smoking		2148 (20.1)	159 (22.3)
Cerebrovascular Disease		1586 (14.8)	167 (23.4)
Solid Organ Transplantation		463 (4.3)	84 (11.8)
Anemia		1859 (17.4)	282 (39.6)
Drug Abuse		1113 (10.4)	75 (10.5)
**In-Hospital Medication or High Acuity Treatment**			
Remdesivir		4702 (44.0)	318 (44.6)
Dexamethasone		5094 (47.6)	287 (40.3)
Oral Blood Thinner		1715 (16.0)	160 (22.4)
Monoclonal Antibodies		317 (3.0)	23 (3.2)
Bamlanivimab		40 (0.4)	6 (0.8)
Casirivimab		113 (1.1)	6 (0.8)
Imdevimab		108 (1.0)	5 (0.7)
Sotrovimab		21 (0.2)	4 (0.6)
Tocilizumab		143 (1.3)	7 (1.0)
SSRIs		1331 (12.4)	106 (14.9)
Fluoxetine		223 (2.1)	19 (2.7)
Citalopram		594 (5.6)	44 (6.2)
Sertraline		439 (4.1)	41 (5.8)
Paroxetine		84 (0.8)	6 (0.8)
Escitalopram		426 (4.0)	30 (4.2)
Vortioxetine		14 (0.1)	1 (0.1)
Mechanical Ventilation		1204 (11.3)	126 (17.7)
ECMO		22 (0.2)	2 (0.3)
Tracheostomy		156 (1.5)	13 (1.8)
**In-Hospital Complication**			
Deep Vein Thrombosis		54 (0.5)	20 (2.8)
Intracerebral Bleeding, Cerebral Infarction, or Stroke		799 (7.5)	81 (11.4)
Lung Edema		585 (5.5)	170 (23.8)
Lung Embolism		571 (5.3)	25 (3.5)
Acute Myocardial Infarction		1193 (11.2)	227 (31.8)
Septic Shock		3687 (34.5)	377 (52.9)
High hospital LOS		5159 (48.2)	424 (59.5)
ICU Stay		1870 (17.5)	129 (18.1)
High ICU LOS		676 (6.3)	42 (5.9)
Hospital Death within 180 Days		112 (1.0)	16 (2.2)
**Other Complication**			
30-Day Readmission		1200 (11.2)	144 (20.2)
Non-Hospital Death within 180 Days		242 (2.3)	19 (2.7)
Overall Death within 180 Days		354 (3.3)	35 (4.9)

Notes: ADI = area deprivation index, CVD = Collagen Vascular Disease, ECMO = extracorporeal membrane oxygenation, HIV/AIDS = human immunodeficiency virus/acquired immune deficiency syndrome, ICU = intensive care unit, IQR = interquartile range, LOS = length of stay, SSRIs = selective serotonin reuptake inhibitors, Transplantation = solid organ transplantation, excluding kidney, Stroke = intracerebral bleeding, cerebral infarction, or stroke, Pulmonary Disease includes asthma and chronic obstructive pulmonary disease.

^1^ADI is calculated in deciles with an ADI of 1 and 10 signifying a neighborhood that is least and most socioeconomically disadvantaged, respectively

We included area deprivation index (ADI) as a factor in our analyses. The ADI for a given neighborhood (census block group) provides a measure of relative socioeconomic disadvantage [11,12] that encompasses income, education, employment, and housing against the mean values in the State (ADIs are state specific). The ADI ranges from 1 to 10, with 1 and 10 signifying a neighborhood that is least and most socioeconomically disadvantaged, respectively. Individuals in the UC CORDS database were assigned an ADI based upon their residence zip code. In our study, 5.3% of those with ESRD and 10.2% of those without ESRD were missing an ADI. Medications administered most likely for COVID-19 treatment to patients during their qualifying hospital stay were also queried, as was information collected on the in-hospital use of high acuity treatments. The number of COVID-19 vaccines recorded for patients in the UC system prior to index hospital admission was queried, however in our study, no record of COVID-19 vaccination was found for 81.5% of those with ESRD and 80.1% of those without ESRD.

We utilized simple and multiple Cox proportional hazards regression models to analyze the association of ESRD status with each of the study outcomes. In addition to unadjusted models, two adjusted Cox proportional hazards regression models were fitted for each of the outcomes hospital, non-hospital, and overall death within 180 days post-discharge, and for 30-day hospital readmission. Model 1 included the demographic variables sex, age per 10 years increase, and race/ethnicity, and also a variable for index hospitalization period. Model 2 included the above demographic variables and the variable for index hospitalization period, plus any comorbid conditions associated at the p = 0.15 level with both the outcome and the study exposure, and which caused a ≥ 10% change in the unadjusted Wald chi-square HR when added as a factor to the unadjusted Cox proportional hazards regression model. For the outcomes hospital, non-hospital, and overall death within 180 days post-discharge model 2 included the comorbid conditions anemia, cancer, cardiac peripheral vascular disease, cardiac arrhythmias, major cardiac disease, cardiac valvular disease, coagulopathy, and hypertension, while for the outcome 30-day hospital readmission model 2 included the comorbid conditions anemia, major cardiac disease, and coagulopathy. We checked the proportional hazard assumption for each of our multivariate Cox proportional hazards regression models by plotting the scaled Schoenfeld residuals, and we did not find any violations of this assumption. Additionally, forward stepwise multivariate Cox proportional hazards regression models were utilized to determine independent risk factors significantly associated with hospital and non-hospital death within 180 days post-discharge for all individuals, and with 30-day readmission, separately among individuals with and without ESRD. Risk factors with Wald chi-square HRs significant at the p = 0.05 level were retained in each of the multivariate models. In all analyses, we used two-sided tests and alpha = 0.05 without adjusting for multiple comparisons.

## Results

After applying the study inclusion and exclusion criteria, a total of 11,406 individuals were discharged alive after being hospitalized for COVID-19 between January 6, 2020 to August 3, 2023. Of these, 713 (6.3%) had ESRD and 10,693 (93.7%) did not. Of the 11,406 individuals, 1,344 (11.8%) were readmitted to the hospital within 30 days post-discharge, 144 (20.2%) in the ESRD group and 1,200 (11.2%) in the non-ESRD group. Additionally, of the 11,406 individuals, 389 (3.4%) died within 180 days post-discharge, 35 (4.9%) in the ESRD group and 354 (3.3%) in the non-ESRD group. Of the 35 individuals with ESRD who died within 180 days post-discharge, 16 (45.7%) died in-hospital, while 112 (31.6%) of the 354 individuals without ESRD who died within 180 days post-discharge did so in-hospital.

The demographic and clinical characteristics for individuals with/without ESRD prior to hospital admission as well as the medications and high acuity treatments administered to them in-hospital are provided in Table 1. Individuals with ESRD were more likely than those who did not have this disease to live in a more socioeconomically disadvantaged census tract, to self-identify as either Hispanic (48.9% vs 36.7%) or Black (12.1% vs 8.0%), to be obese (38.1% vs 28.0%) to have more comorbid conditions, to receive an oral blood thinner (22.4% vs 16.0%), to receive mechanical ventilation (17.7% vs 11.3%), to have one or more complications during their qualifying hospital stay, and for that stay to be ≥ 7 days (59.5% vs 48.2%). In contrast, individuals with ESRD were less likely than those without ESRD to receive dexamethasone during their qualifying hospital stay (40.3% vs 47.6%).

**Fig 1:**
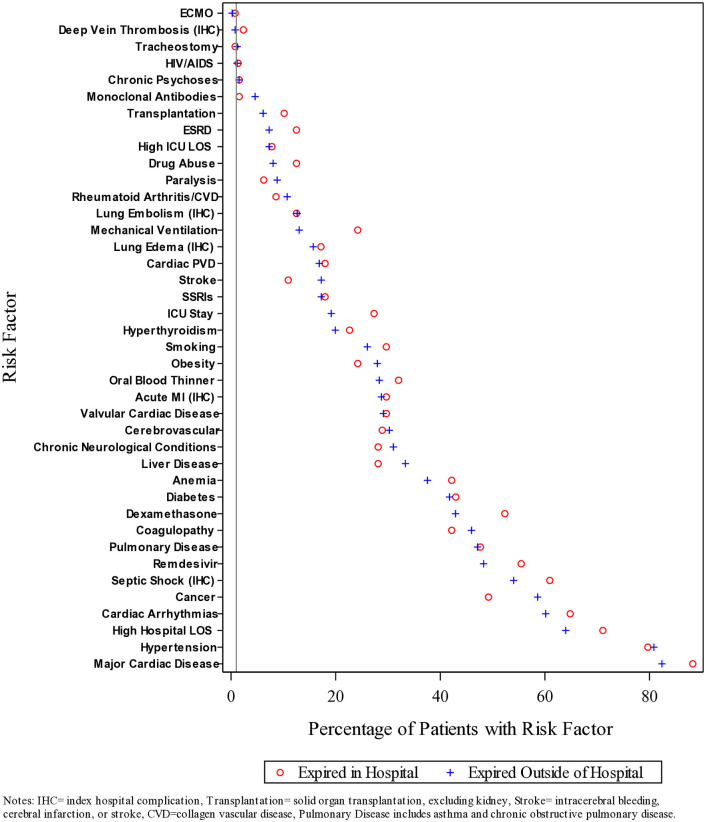
Percentage of individuals who died in-hospital/outside of hospital within 180 days post-hospital discharge with selected demographic and clinical risk factors (N = 11,406). Notes: IHC = index hospital complication, Transplantation = solid organ transplantation, excluding kidney, Stroke = intracerebral bleeding cerebral infraction or stroke. CVD = collagen vascular disease, Pulmonary disease includes asthma and chronic obstructive pulmonary disease.

The baseline characteristics of those who died in-hospital/outside of hospital within 180 days post-discharge, and the medications and treatments administered to them during their index in-hospital stay, are provided in Table 2. Of the 11,406 patients who were discharged alive, 128 (1.1%) died in-hospital within 180 days. Those who died in-hospital were older (median age 68 vs 60), more likely to be smokers (29.7% vs 20.1%), to have more comorbid conditions, and to receive Remdesivir (55.5% vs 43.9%), dexamethasone (52.3% vs 47.1%), and an oral blood thinner (32.0% vs 16.3%), to be on a ventilator (24.2% vs 11.5%), to have more complications, to be admitted to an ICU (27.3% vs 17.4%), and to remain in the hospital ≥7 days (71.1% vs 48.7%) during their qualifying hospital stay. Of the 11,406 patients who were discharged alive, 261 (2.3%) died outside of the hospital within 180 days. Those who died outside of the hospital were older (median age 70 vs 60), more likely to self-identify as White (43.3% vs 29.8%), to be smokers (26.1% vs 20.1%), to have more comorbid conditions, to receive an oral blood thinner (28.4% vs 16.2), to have more complications, and to remain in the hospital ≥7 days (64.0% vs 48.6%) during their qualifying hospital stay. In contrast, these individuals were less likely than those without ESRD to self-identify as Hispanic (25.7% vs 37.7%). The percentages of those who died in-hospital/outside of hospital within 180 days post-hospital discharge with selected demographic and clinical risk factors are shown in Fig 1.

**Table 2:** Demographic and clinical characteristics of individuals who died in-hospital/outside of hospital 180 days post-hospital discharge (N = 11,406).

		Died In-Hospital		Died Outside of Hospital	
Variable		No (N = 11,278)	Yes (N = 128)	No (N = 11,145)	Yes (N = 261)
Age (years), median, (IQR)		60 (44,73)	68 (58,78.5)	60 (44,73)	70 (58,83)
Age (%)	18–39	2178 (19.3)	8 (6.3)	2175 (19.5)	11 (4.2)
	40–49	1345 (11.9)	8 (6.3)	1333 (12)	20 (7.7)
	50–59	1947 (17.3)	21 (16.4)	1924 (17.3)	44 (16.9)
	60–69	2233 (19.8)	35 (27.3)	2214 (19.9)	54 (20.7)
	70–79	1755 (15.6)	25 (19.5)	1733 (15.5)	47 (18.0)
	80+	1820 (16.1)	31 (24.2)	1766 (15.8)	85 (32.6)
Sex	Male	6129 (54.3)	81 (63.3)	6071 (54.5)	139 (53.3)
Race/Ethnicity	Asian American	1358 (12.0)	17 (13.3)	1344 (12.1)	31 (11.9)
	Black	925 (8.2)	14 (10.9)	915 (8.2)	24 (9.2)
	Hispanic	4226 (37.5)	44 (34.4)	4203 (37.7)	67 (25.7)
	Other/Unknown	1383 (12.3)	10 (7.8)	1367 (12.3)	26 (10.0)
	White	3386 (30.0)	43 (33.6)	3316 (29.8)	113 (43.3)
Hospitalization Period	Jan-May 2020	379 (3.4)	1 (0.8)	370 (3.3)	10 (3.8)
	Jun-Aug 2020	1054 (9.3)	7 (5.5)	1048 (9.4)	13 (5.0)
	Sep 2020-Jan 2021	3052 (27.1)	42 (32.8)	3030 (27.2)	64 (24.5)
	Feb-May 2021	575 (5.1)	3 (2.3)	568 (5.1)	10 (3.8)
	Jun-Nov 2021	951 (8.4)	8 (6.3)	933 (8.4)	26 (10.0)
	Dec 2021-Feb 2022	1658 (14.7)	20 (15.6)	1636 (14.7)	42 (16.1)
	Mar-Sep 2022	1656 (14.7)	22 (17.2)	1625 (14.6)	53 (20.3)
	Oct 2022-Aug 2023	1953 (17.3)	25 (19.5)	1935 (17.4)	43 (16.5)
COVID-19 Vaccinations Prior to Index Hospitalization	Missing	9051 (80.3)	90 (70.3)	8965 (80.4)	176 (67.4)
	1	227 (2.0)	5 (3.9)	223 (2.0)	9 (3.4)
	2	674 (6.0)	14 (10.9)	667 (6.0)	21 (8.0)
	3	661 (5.9)	14 (10.9)	642 (5.8)	33 (12.6)
	4	0	0	300 (2.7)	16 (6.1)
	≥5	349 (3.1)	5 (3.9)	348 (3.1)	6 (2.3)
ADI^1^	Missing	1115 (9.9)	10 (7.8)	1109 (10.0)	16 (6.1)
	1	1410 (12.5)	16 (12.5)	1388 (12.5)	38 (14.6)
	2	1025 (9.1)	8 (6.3)	1002 (9.0)	31 (11.9)
	3	1044 (9.3)	11 (8.6)	1030 (9.2)	25 (9.6)
	4	1223 (10.8)	11 (8.6)	1208 (10.8)	26 (10.0)
	5	1237 (11.0)	10 (7.8)	1212 (10.9)	35 (13.4)
	6	1173 (10.4)	18 (14.1)	1165 (10.5)	26 (10.0)
	7	1017 (9.0)	18 (14.1)	1018 (9.1)	17 (6.5)
	8	836 (7.4)	8 (6.3)	828 (7.4)	16 (6.1)
	9	637 (5.6)	7 (5.5)	625 (5.6)	19 (7.3)
	10	561 (5.0)	11 (8.6)	560 (5.0)	12 (4.6)
**Comorbid Condition**					
Major Cardiac Disease		6375 (56.5)	113 (88.3)	6273 (56.3)	215 (82.4)
Valvular Cardiac Disease		1576 (14.0)	38 (29.7)	1538 (13.8)	76 (29.1)
Cardiac Arrhythmias		4519 (40.1)	83 (64.8)	4445 (39.9)	157 (60.2)
Cardiac Peripheral Vascular Disease		1120 (9.9)	23 (18.0)	1099 (9.9)	44 (16.9)
Coagulopathy		2726 (24.2)	54 (42.2)	2660 (23.9)	120 (46.0)
Cancer		2225 (19.7)	63 (49.2)	2135 (19.2)	153 (58.6)
Diabetes		4245 (37.6)	55 (43.0)	4191 (37.6)	109 (41.8)
HIV/AIDS		0	0	149 (1.3)	3 (1.1)
Hypertension		7024 (62.3)	102 (79.7)	6915 (62.0)	211 (80.8)
Hyperthyroidism		1725 (15.3)	29 (22.7)	1702 (15.3)	52 (19.9)
Liver Disease		1957 (17.4)	36 (28.1)	1906 (17.1)	87 (33.3)
Chronic Neurological Conditions		2169 (19.2)	36 (28.1)	2124 (19.1)	81 (31.0)
Obesity		3238 (28.7)	31 (24.2)	3196 (28.7)	73 (28.0)
Paralysis		509 (4.5)	8 (6.3)	494 (4.4)	23 (8.8)
Chronic Psychoses		466 (4.1)	2 (1.6)	464 (4.2)	4 (1.5)
Pulmonary Disease		3497 (31.0)	61 (47.7)	3435 (30.8)	123 (47.1)
Rheumatoid Arthritis/CVD		792 (7.0)	11 (8.6)	775 (7.0)	28 (10.7)
Smoking		2269 (20.1)	38 (29.7)	2239 (20.1)	68 (26.1)
Cerebrovascular		1716 (15.2)	37 (28.9)	1674 (15.0)	79 (30.3)
Solid Organ Transplantation, excluding Kidney		534 (4.7)	13 (10.2)	531 (4.8)	16 (6.1)
Anemia		2087 (18.5)	54 (42.2)	2043 (18.3)	98 (37.5)
Drug Abuse		1172 (10.4)	16 (12.5)	1167 (10.5)	21 (8.0)
**In-Hospital Medication or High Acuity Treatment**					
Remdesivir		4949 (43.9)	71 (55.5)	4894 (43.9)	126 (48.3)
Dexamethasone		5314 (47.1)	67 (52.3)	5269 (47.3)	112 (42.9)
Oral Blood Thinner		1834 (16.3)	41 (32.0)	1801 (16.2)	74 (28.4)
Monoclonal Antibodies		338 (3.0)	2 (1.6)	328 (2.9)	12 (4.6)
Casirivimab		118 (1.0)	1 (0.8)	114 (1.0)	5 (1.9)
Imdevimab		112 (1.0)	1 (0.8)	108 (1.0)	5 (1.9)
Sotrovimab		0	0	22 (0.2)	3 (1.1)
Tocilizumab		149 (1.3)	1 (0.8)	146 (1.3)	4 (1.5)
SSRIs		1414 (12.5)	23 (18)	1392 (12.5)	45 (17.2)
Fluoxetine		237 (2.1)	5 (3.9)	236 (2.1)	6 (2.3)
Citalopram		627 (5.6)	11 (8.6)	617 (5.5)	21 (8.0)
Sertraline		474 (4.2)	6 (4.7)	462 (4.1)	18 (6.9)
Paroxetine		0	0	89 (0.8)	1 (0.4)
Escitalopram		448 (4.0)	8 (6.3)	443 (4.0)	13 (5.0)
Vortioxetine		14 (0.1)	1 (0.8)		
Mechanical Ventilation		1299 (11.5)	31 (24.2)	1296 (11.6)	34 (13.0)
ECMO		23 (0.2)	1 (0.8)		
Tracheostomy		168 (1.5)	1 (0.8)	166 (1.5)	3 (1.1)
**In-Hospital Complication**					
Deep Vein Thrombosis		71 (0.6)	3 (2.3)	72 (0.6)	2 (0.8)
Intracerebral Bleeding, Cerebral Infarction, or Stroke		866 (7.7)	14 (10.9)	835 (7.5)	45 (17.2)
Lung Edema		733 (6.5)	22 (17.2)	714 (6.4)	41 (15.7)
Lung Embolism		580 (5.1)	16 (12.5)	563 (5.1)	33 (12.6)
Acute Myocardial Infarction		1382 (12.3)	38 (29.7)	1345 (12.1)	75 (28.7)
Septic Shock		3986 (35.3)	78 (60.9)	3923 (35.2)	141 (54.0)
High Hospital LOS		5492 (48.7)	91 (71.1)	5416 (48.6)	167 (64.0)
ICU Stay		1964 (17.4)	35 (27.3)	1949 (17.5)	50 (19.2)
High ICU LOS		708 (6.3)	10 (7.8)	699 (6.3)	19 (7.3)
ESRD		697 (6.2)	16 (12.5)	694 (6.2)	19 (7.3)

Notes: ADI = area deprivation index, CVD = Collagen Vascular Disease, ECMO = extracorporeal membrane oxygenation, HIV/AIDS = human immunodeficiency virus/acquired immune deficiency syndrome, ICU = intensive care unit, IQR = interquartile range, LOS = length of stay, SSRIs = selective serotonin reuptake inhibitors, Transplantation = solid organ transplantation, excluding kidney, Stroke = intracerebral bleeding, cerebral infarction, or stroke, Pulmonary Disease includes asthma and chronic obstructive pulmonary disease.

^1^ADI is calculated in deciles with an ADI of 1 and 10 signifying a neighborhood that is least and most socioeconomically disadvantaged, respectively

**Fig 2:**
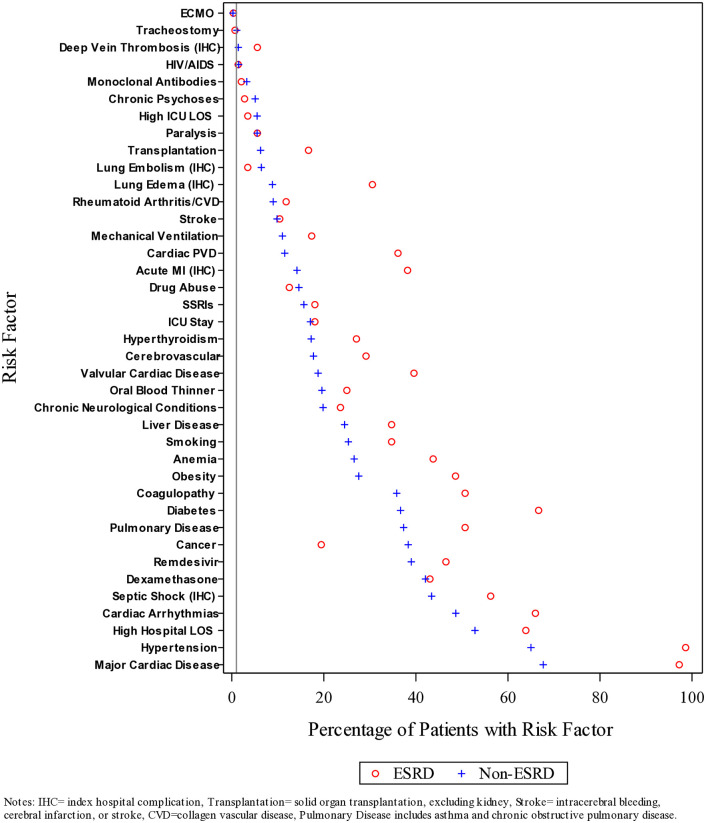
Percentage of individuals with/without ESRD readmitted within 30 days post-hospital discharge with selected demographic and clinical risk factors (N = 11,406). Notes: IHC = index hospital complication, Transplantation = solid organ transplantation, excluding kidney, Stroke = intracerebral bleeding cerebral infraction or stroke. CVD = collagen vascular disease, Pulmonary disease includes asthma and chronic obstructive pulmonary disease.

The baseline characteristics of those with/without ESRD, and the medications and treatments administered to them in the hospital, by readmission status 30 days post-discharge, are provided in Table 3. Of the 713 patients with ESRD who were discharged alive, 144 (20.2%) were readmitted to the hospital within 30 days. These individuals were more likely to be female (50.0% vs 42.4%), to self-identify as Hispanic (52.8% vs 48.0%) or Black (15.3% vs 11.2%), to be smokers (34.7% vs 19.2%), to be obese (48.6% vs 35.5%), to have more comorbid conditions, and to receive an SSRI (18.1% vs 14.1%), to have more in-hospital complications, and to remain in the hospital ≥7 days (63.9% vs 58.3%) during their qualifying hospital stay. Of the 10,693 patients without ESRD who were discharged alive, 1,200 (11.2%) were readmitted to the hospital within 30 days. These individuals were more likely to live in an area with a high ADI, to be smokers (25.3% vs 19.4%), to have more comorbid conditions, and to have more in-hospital complications and remain in the hospital ≥7 days (52.8% vs 47.7%) during their qualifying hospital stay. These individuals were also less likely to have received dexamethasone during that hospital stay (42.1% vs 48.3%). The percentages of individuals with/without ESRD who were readmitted within 30 days post-hospital discharge with selected demographic and clinical risk factors is shown in Fig 2. This figure shows that individuals with ESRD who were readmitted to the hospital had a much higher prevalence of many comorbid conditions than those without ESRD.

**Table 3:** Demographic and clinical characteristics of individuals with/without ESRD by readmission status 30 days post-hospital discharge (N = 11,406).

		Individuals with ESRD		Individuals without ESRD	
Variable		Not Readmitted (N = 569)	Readmitted (N = 144)	Not Readmitted (N = 9,493)	Readmitted (N = 1,200)
Age (years), median, (IQR)		61 (49,71)	62 (51,72)	60 (44,74)	60 (42,73)
Age (%)	18–39	84 (14.8)	18 (12.5)	1820 (19.2)	264 (22.0)
	40–49	65 (11.4)	13 (9.0)	1139 (12.0)	136 (11.3)
	50–59	109 (19.2)	31 (21.5)	1644 (17.3)	184 (15.3)
	60–69	152 (26.7)	37 (25.7)	1832 (19.3)	247 (20.6)
	70–79	91 (16.0)	30 (20.8)	1481 (15.6)	178 (14.8)
	80+	68 (12.0)	15 (10.4)	1577 (16.6)	191 (15.9)
Race/Ethnicity	Asian American	70 (12.3)	10 (6.9)	1143 (12.0)	152 (12.7)
	Black	64 (11.2)	22 (15.3)	746 (7.9)	107 (8.9)
	Hispanic	273 (48)	76 (52.8)	3521 (37.1)	400 (33.3)
	Other/Unknown	59 (10.4)	13 (9.0)	1187 (12.5)	134 (11.2)
	White	103 (18.1)	23 (16.0)	2896 (30.5)	407 (33.9)
Hospitalization Period	Jan-May 2020	21 (3.7)	3 (2.1)	340 (3.6)	16 (1.3)
	Jun-Aug 2020	54 (9.5)	14 (9.7)	918 (9.7)	75 (6.3)
	Sep 2020-Jan 2021	166 (29.2)	34 (23.6)	2627 (27.7)	267 (22.3)
	Feb-May 2021	20 (3.5)	7 (4.9)	486 (5.1)	65 (5.4)
	Jun-Nov 2021	24 (4.2)	10 (6.9)	835 (8.8)	90 (7.5)
	Dec 2021-Feb 2022	112 (19.7)	29 (20.1)	1339 (14.1)	198 (16.5)
	Mar-Sep 2022	65 (11.4)	26 (18.1)	1349 (14.2)	238 (19.8)
	Oct 2022-Aug 2023	107 (18.8)	21 (14.6)	1599 (16.8)	251 (20.9)
COVID-19 Vaccinations Prior to Index Hospitalization	Missing	461 (81.0)	120 (83.3)	7670 (80.8)	890 (74.2)
	1	0	0	193 (2.0)	34 (2.8)
	2	33 (5.8)	11 (7.6)	546 (5.8)	98 (8.2)
	3	35 (6.2)	5 (3.5)	533 (5.6)	102 (8.5)
	4	14 (2.5)	4 (2.8)	254 (2.7)	44 (3.7)
	≥5	21 (3.7)	4 (2.8)	297 (3.1)	32 (2.7)
ADI^1^	Missing	29 (5.1)	9 (6.3)	976 (10.3)	111 (9.3)
	1	43 (7.6)	12 (8.3)	1223 (12.9)	148 (12.3)
	2	43 (7.6)	9 (6.3)	875 (9.2)	106 (8.8)
	3	48 (8.4)	9 (6.3)	890 (9.4)	108 (9.0)
	4	55 (9.7)	21 (14.6)	1043 (11.0)	115 (9.6)
	5	71 (12.5)	11 (7.6)	1035 (10.9)	130 (10.8)
	6	86 (15.1)	21 (14.6)	959 (10.1)	125 (10.4)
	7	69 (12.1)	17 (11.8)	847 (8.9)	102 (8.5)
	8	61 (10.7)	11 (7.6)	682 (7.2)	90 (7.5)
	9	33 (5.8)	9 (6.3)	511 (5.4)	91 (7.6)
	10	31 (5.4)	15 (10.4)	452 (4.8)	74 (6.2)
**Comorbid Condition**					
Major Cardiac Disease		511 (89.8)	140 (97.2)	5025 (52.9)	812 (67.7)
Valvular Cardiac Disease		147 (25.8)	57 (39.6)	1185 (12.5)	225 (18.8)
Cardiac Arrhythmias		307 (54.0)	95 (66.0)	3616 (38.1)	584 (48.7)
Cardiac Peripheral Vascular Disease		151 (26.5)	52 (36.1)	802 (8.4)	138 (11.5)
Coagulopathy		244 (42.9)	73 (50.7)	2033 (21.4)	430 (35.8)
Cancer		80 (14.1)	28 (19.4)	1720 (18.1)	460 (38.3)
Diabetes		395 (69.4)	96 (66.7)	3369 (35.5)	440 (36.7)
HIV/AIDS		11 (1.9)	2 (1.4)	121 (1.3)	18 (1.5)
Hypertension		548 (96.3)	142 (98.6)	5656 (59.6)	780 (65.0)
Hyperthyroidism		130 (22.8)	39 (27.1)	1378 (14.5)	207 (17.3)
Liver Disease		139 (24.4)	50 (34.7)	1510 (15.9)	294 (24.5)
Chronic Neurological Conditions		129 (22.7)	34 (23.6)	1804 (19.0)	238 (19.8)
Obesity		202 (35.5)	70 (48.6)	2666 (28.1)	331 (27.6)
Paralysis		29 (5.1)	8 (5.6)	414 (4.4)	66 (5.5)
Chronic Psychoses		20 (3.5)	4 (2.8)	383 (4.0)	61 (5.1)
Pulmonary Disease		222 (39.0)	73 (50.7)	2815 (29.7)	448 (37.3)
Rheumatoid Arthritis/CVD		57 (10.0)	17 (11.8)	621 (6.5)	108 (9.0)
Smoking		109 (19.2)	50 (34.7)	1844 (19.4)	304 (25.3)
Cerebrovascular		125 (22.0)	42 (29.2)	1373 (14.5)	213 (17.8)
Solid Organ Transplantation, excluding Kidney		60 (10.5)	24 (16.7)	388 (4.1)	75 (6.3)
Anemia		219 (38.5)	63 (43.8)	1540 (16.2)	319 (26.6)
Drug Abuse		57 (10.0)	18 (12.5)	938 (9.9)	175 (14.6)
**In-Hospital Medication or High Acuity Treatment**					
Remdesivir		251 (44.1)	67 (46.5)	4234 (44.6)	468 (39.0)
Dexamethasone		225 (39.5)	62 (43.1)	4589 (48.3)	505 (42.1)
Oral Blood Thinner		124 (21.8)	36 (25.0)	1480 (15.6)	235 (19.6)
Monoclonal Antibodies		20 (3.5)	3 (2.1)	278 (2.9)	39 (3.3)
Bamlanivimab		5 (0.9)	1 (0.7)	36 (0.4)	4 (0.3)
Casirivimab		5 (0.9)	1 (0.7)	96 (1.0)	17 (1.4)
Imdevimab		0	0	92 (1.0)	16 (1.3)
Sotrovimab		3 (0.5)	1 (0.7)	13 (0.1)	8 (0.7)
Tocilizumab		0	0	133 (1.4)	10 (0.8)
SSRIs		80 (14.1)	26 (18.1)	1143 (12.0)	188 (15.7)
Fluoxetine		14 (2.5)	5 (3.5)	191 (2.0)	32 (2.7)
Fluvoxamine		0	0	16 (0.2)	1 (0.1)
Citalopram		37 (6.5)	7 (4.9)	506 (5.3)	88 (7.3)
Sertraline		30 (5.3)	11 (7.6)	378 (4.0)	61 (5.1)
Paroxetine		3 (0.5)	3 (2.1)	74 (0.8)	10 (0.8)
Escitalopram		27 (4.7)	3 (2.1)	370 (3.9)	56 (4.7)
Vortioxetine		0	0	11 (0.1)	3 (0.3)
Mechanical Ventilation		101 (17.8)	25 (17.4)	1072 (11.3)	132 (11.0)
ECMO		0	0	19 (0.2)	3 (0.3)
Tracheostomy		12 (2.1)	1 (0.7)	143 (1.5)	13 (1.1)
**In-Hospital Complication**					
Deep Vein Thrombosis		12 (2.1)	8 (5.6)	37 (0.4)	17 (1.4)
Intracerebral Bleeding, Cerebral Infarction, or Stroke		66 (11.6)	15 (10.4)	681 (7.2)	118 (9.8)
Lung Edema		126 (22.1)	44 (30.6)	479 (5.0)	106 (8.8)
Lung Embolism		20 (3.5)	5 (3.5)	494 (5.2)	77 (6.4)
Acute Myocardial Infarction		172 (30.2)	55 (38.2)	1023 (10.8)	170 (14.2)
Septic Shock		296 (52.0)	81 (56.3)	3166 (33.4)	521 (43.4)
High Hospital LOS		332 (58.3)	92 (63.9)	4525 (47.7)	634 (52.8)
ICU Stay		103 (18.1)	26 (18.1)	1665 (17.5)	205 (17.1)
High ICU LOS		37 (6.5)	5 (3.5)	610 (6.4)	66 (5.5)

Notes: ADI = area deprivation index, CVD = Collagen Vascular Disease, ECMO = extracorporeal membrane oxygenation, HIV/AIDS = human immunodeficiency virus/acquired immune deficiency syndrome, ICU = intensive care unit, IQR = interquartile range, LOS = length of stay, SSRIs = selective serotonin reuptake inhibitors, Transplantation = solid organ transplantation, excluding kidney, Stroke = intracerebral bleeding, cerebral infarction, or stroke, Pulmonary Disease includes asthma and chronic obstructive pulmonary disease.

^1^ADI is calculated in deciles with an ADI of 1 and 10 signifying a neighborhood that is least and most socioeconomically disadvantaged, respectively

Unadjusted and multivariable-adjusted HRs for hospital, non-hospital, and overall death within 180 days post-discharge, and for 30-day hospital readmission among those with and without ESRD are provided in Table 4. After determining the factors to be included in our adjusted model 2 analyses, we found that hospital death and overall death within 180 days post-discharge were significantly higher for individuals with ESRD in the unadjusted analyses but not in the analyses adjusted for demographic variables, hospitalization period, and comorbid conditions (adjusted HR 1.36, 95% CI 0.78–2.37 and adjusted HR 1.05, 95% CI 0.73–1.51, respectively). We did not find ESRD status to be associated with non-hospital death within 180 days post-discharge in either the unadjusted or the adjusted analyses (adjusted HR 0.89, 95% CI 0.55–1.44). Finally, we found that compared to those without kidney failure, those with ESRD had a significantly higher hazard of 30-day readmission post-discharge in both the unadjusted and the adjusted analyses (adjusted HR 1.36, 95% CI 1.14–1.63, p = 0.001).

**Table 4:** Hazard ratios for outcomes post-hospital discharge among individuals with/without ESRD (N = 11,406) (the group without ESRD is the referent).

Outcomes	HR	95% CI	p Value
**Hospital Death within 180 days post-hospital discharge**			
Unadjusted	2.15	1.27, 3.63	0.004
Adjusted model 1^**1**^	2.07	1.22, 3.52	0.007
Adjusted model 2^**2**^	1.36	0.78, 2.37	0.278
**Non-Hospital Death within 180 days post-hospital discharge**			
Unadjusted	1.18	0.74, 1.88	0.495
Adjusted model 1^**1**^	1.25	0.78, 1.99	0.360
Adjusted model 2^**2**^	0.89	0.55, 1.44	0.634
**Overall Death within 180 days post-hospital discharge**			
Unadjusted	1.49	1.05, 2.11	0.024
Adjusted model 1^**1**^	1.53	1.08, 2.17	0.017
Adjusted model 2^**2**^	1.05	0.73, 1.51	0.795
**30-Day Readmission post-hospital discharge**			
Unadjusted	1.86	1.57, 2.21	< 0.001
Adjusted model 1^**1**^	1.90	1.60, 2.26	< 0.001
Adjusted model 2^**3**^	1.36	1.14, 1.63	0.001

Note: HR = Wald chi-square hazard ratio, CI = Wald confidence interval.

Covariate adjustment in multivariate-adjusted regression models was performed in a sequential manner as follows:

1 Adjusted model 1 was adjusted for the demographic variables sex, age in years, and race/ethnicity, and for the index hospitalization period

2 Adjusted model 2 was adjusted for demographic variables, index hospitalization period, anemia, cancer, cardiac peripheral vascular disease, cardiac arrhythmias, major cardiac disease, cardiac valvular disease, coagulopathy, and hypertension

3 Adjusted model 2 was adjusted for demographic variables, index hospitalization period, anemia, major cardiac disease, and coagulopathy

**Fig 3:**
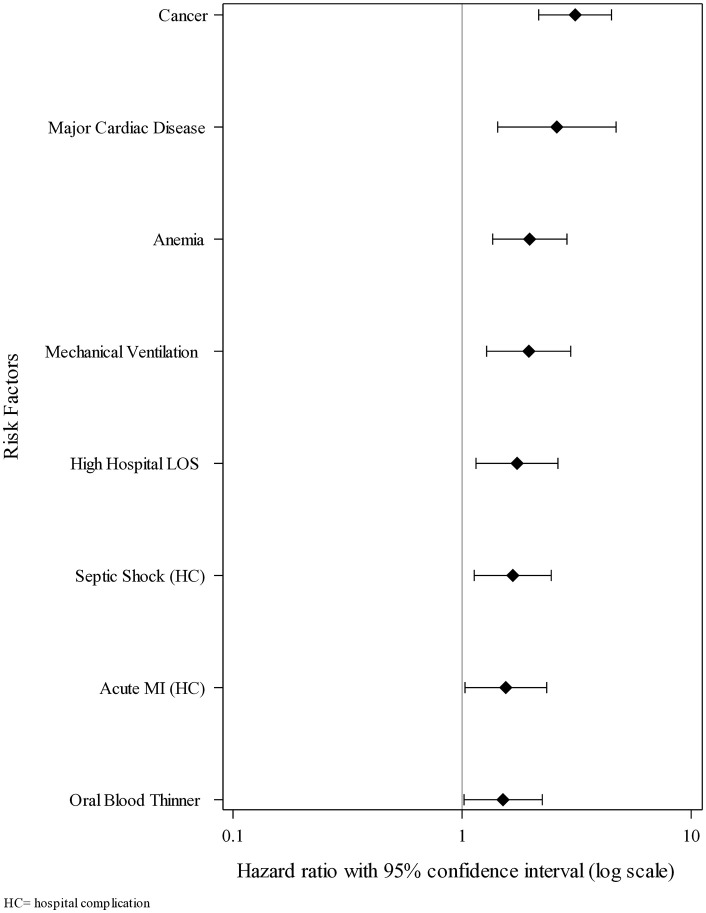
Forest plot showing the multivariate risk factors for hospital death within 180 days post-hospital discharge (N = 11,406). HC = hospital complication.

**Fig 4:**
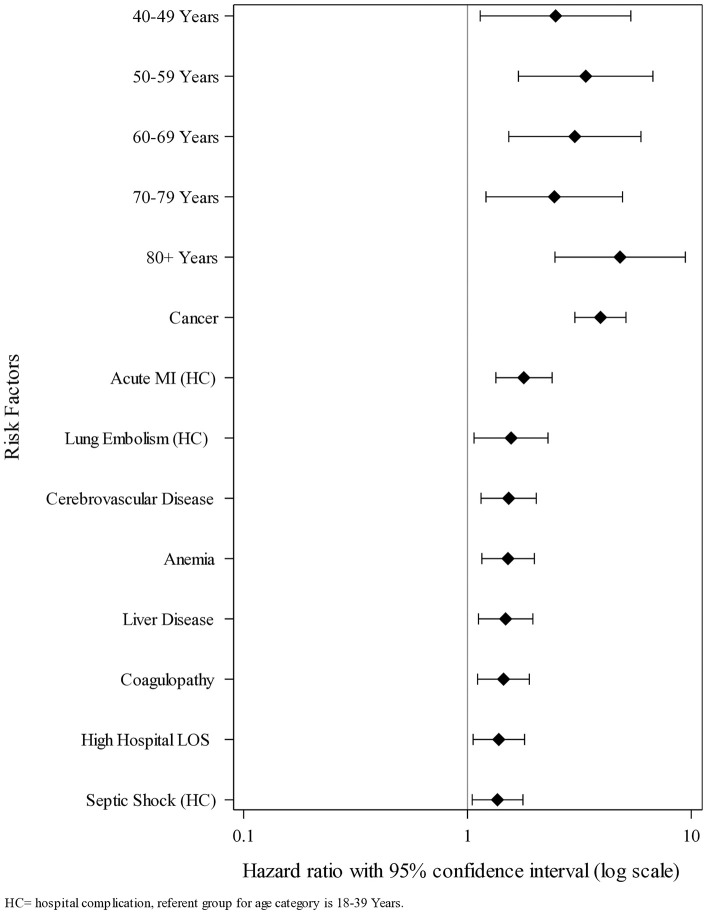
Forest plot showing the multivariate risk factors for non-hospital death within 180 days post-hospital discharge (N = 11,406). HC = hospital complication, referent group for age category is 18–39 years.

The analysis of risk factors for hospital death within 180 days post-discharge is shown in Table 5, with multivariate risk factors for this outcome shown in Fig 3. We found cancer to be the factor most associated with a significantly increased risk of hospital death within 180 days post-discharge (adjusted HR = 3.12, 95% CI 2.16–4.49, p < 0.0001), followed by major cardiac disease (adjusted HR = 2.59, 95% CI 1.43–4.7, p = 0.0017), anemia (adjusted HR = 1.97, 95% CI 1.36–2.87, p = 0.0004), mechanical ventilation (adjusted HR = 1.96, 95% CI 1.28–2.98, p = 0.0018), high hospital length of stay (adjusted HR = 1.74, 95% CI 1.15–2.62, p = 0.0082), the hospital complications septic shock (adjusted HR = 1.67, 95% CI 1.13–2.45, p = 0.0093), and acute MI (adjusted HR = 1.55, 95% CI 1.03–2.34, p = 0.0364), and the administration of oral blood thinners during the index hospitalization (adjusted HR = 1.51, 95% CI 1.02–2.24, p = 0.0409). The analysis of risk factors for non-hospital death within 180 days post-discharge is shown in Table 6 with multivariate risk factors for this outcome shown in Fig 4. We found being 80 or more years of age to be the factor most associated with a significantly increased risk of non-hospital death within 180 days post-discharge (adjusted HR = 4.81, 95% CI 2.46–9.41, p < 0.0001), followed by cancer (adjusted HR = 3.93, 95% CI 3.02–5.11, p < 0.0001), being age 50–59 years (adjusted HR = 3.38, 95% CI 1.69–6.74, p = 0.0005), being age 60–69 years (adjusted HR = 3.02, 95% CI 1.53–5.96, p = 0.0015), being age 40–49 years (adjusted HR = 2.48, 95% CI 1.14–5.37, p = 0.0215), being age 70–79 years (adjusted HR = 2.45, 95% CI 1.21–4.93, p = 0.0125), the hospital complications acute myocardial infarction (adjusted HR = 1.79, 95% CI 1.34–2.39, p < 0.0001) and lung embolism (adjusted HR = 1.57, 95% CI 1.07–2.29, p = 0.0211), cerebrovascular disease (adjusted HR = 1.53, 95% CI 1.15–2.03, p = 0.0031), anemia (adjusted HR = 1.52, 95% CI 1.16–1.99, p = 0.0026), liver disease (adjusted HR = 1.48, 95% CI 1.12–1.96, p = 0.0064), coagulopathy (adjusted HR = 1.45, 95% CI 1.11–1.89, p = 0.0065), high hospital length of stay (adjusted HR = 1.38, 95% CI 1.06–1.80, p = 0.0173), and the hospital complication septic shock (adjusted HR = 1.36, 95% CI 1.05–1.77, p = 0.0207).

**Table 5:** Univariate and multivariate Cox proportional hazards regression analyses of risk factors associated with hospital death within 180 days post-hospital discharge (N = 11,406, Number of Hospital Deaths = 128).

			Univariate			Multivariate^1^		
Variable		Hospital Deaths	HR	95% CI	p Value	Adjusted HR	95% CI	p Value
Age Group	18-39 (referent)							
	40-49	8	1.62	0.61, 4.31	0.3369			
	50-59	21	2.92	1.30, 6.60	0.0098			
	60-69	35	4.24	1.97, 9.15	0.0002			
	70-79	25	3.85	1.74, 8.54	0.0009			
	80+	31	4.61	2.12, 10.04	0.0001			
Sex	Female (referent)							
	Male	81	1.44	1.01, 2.07	0.0448			
Race/Ethnicity	White (referent)							
	Asian American	17	0.99	0.56, 1.73	0.9613			
	Black	14	1.19	0.65, 2.18	0.5693			
	Hispanic	44	0.82	0.54, 1.25	0.3585			
	Other/Unknown	10	0.57	0.29, 1.14	0.1106			
Hospitalization Period	Jan-May 2020	1	0.51	0.05, 4.88	0.5568			
	Jun-Aug 2020	7	1.27	0.33, 4.92	0.7262			
	Sep 2020-Jan 2021	42	2.63	0.82, 8.49	0.1055			
	Feb-May 2021 (referent)							
	Jun-Nov 2021	8	1.61	0.43, 6.07	0.4813			
	Dec 2021-Feb 2022	20	2.31	0.69, 7.76	0.1774			
	Mar-Sep 2022	22	2.53	0.76, 8.47	0.1308			
	Oct 2022-Aug 2023	25	2.44	0.74, 8.10	0.1435			
ADI^**2**^			1.06	0.99, 1.13	0.0837			
**Comorbid Condition** ^**3**^								
Kidney Failure		16	2.15	1.27, 3.63	0.0042			
Major Cardiac Disease		113	5.75	3.36, 9.85	<.0001	2.59	1.43, 4.70	0.0017
Valvular Cardiac Disease		38	2.58	1.76, 3.77	<.0001			
Cardiac Arrhythmias		83	2.74	1.91, 3.94	<.0001			
Peripheral Vascular Disease		23	1.98	1.26, 3.10	0.0031			
Coagulopathy		54	2.27	1.60, 3.23	<.0001			
Anemia		54	3.18	2.24, 4.52	<.0001	1.97	1.36, 2.87	0.0004
Cancer		63	3.90	2.76, 5.51	<.0001	3.12	2.16, 4.49	<.0001
Diabetes		55	1.25	0.88, 1.77	0.2173			
Hypertension		102	2.37	1.54, 3.64	<.0001			
Hyperthyroidism		29	1.61	1.07, 2.44	0.0232			
Chronic Neurological Conditions		36	1.64	1.12, 2.41	0.0119			
Obesity		31	0.79	0.53, 1.19	0.2647			
Paralysis		8	1.41	0.69, 2.88	0.3495			
Chronic Psychoses		2	0.37	0.09, 1.49	0.1626			
Pulmonary Disease		61	2.01	1.42, 2.85	<.0001			
Rheumatoid Arthritis/CVD		11	1.24	0.67, 2.30	0.4924			
Smoking		38	1.67	1.14, 2.44	0.0081			
Solid Organ Transplantation		13	2.26	1.27, 4.01	0.0053			
Liver Disease		36	1.85	1.26, 2.73	0.0017			
Cerebrovascular Disease		37	2.25	1.54, 3.30	<.0001			
Drug Abuse		16	1.23	0.73, 2.08	0.4378			
**In-Hospital Medication or High Acuity Treatment**								
Remdesivir		71	1.59	1.12, 2.25	0.0094			
Dexamethasone		67	1.23	0.87, 1.74	0.2382			
Oral Blood Thinner		41	2.41	1.66, 3.49	<.0001	1.51	1.02, 2.24	0.0409
Monoclonal Antibodies		2	0.52	0.13, 2.09	0.3530			
SSRIs		23	1.52	0.97, 2.39	0.0680			
Mechanical Ventilation		31	2.44	1.63, 3.65	<.0001	1.96	1.28, 2.98	0.0018
ECMO		1	3.81	0.53, 27.25	0.1827			
Tracheostomy		1	0.52	0.07, 3.73	0.5165			
**In-Hospital Complication**1								
Deep Vein Thrombosis		3	3.70	1.18, 11.63	0.0251			
Intracerebral Bleeding/Cerebral Infarction/Stroke		14	1.48	0.85, 2.57	0.1694			
Lung Edema		22	2.95	1.86, 4.67	<.0001			
Lung Embolism		16	2.62	1.55, 4.42	0.0003			
Acute Myocardial Infarction		38	2.99	2.05, 4.37	<.0001	1.55	1.03, 2.34	0.0364
Septic Shock		78	2.83	1.99, 4.04	<.0001	1.67	1.13, 2.45	0.0093
High Hospital LOS		91	2.58	1.76, 3.78	<.0001	1.74	1.15, 2.62	0.0082
ICU Stay		35	1.78	1.21, 2.62	0.0037			
High ICU LOS		10	1.26	0.66, 2.41	0.4765			

Abbreviations: ADI = area deprivation index, CI = Wald confidence interval, CVD = collagen vascular disease, ECMO = extracorporeal membrane oxygenation, HR = Wald chi-square hazard ratio, ICU = intensive care unit, LOS = length of stay, SSRIs = selective serotonin reuptake inhibitors, Transplantation = solid organ transplantation, excluding kidney, Stroke = intracerebral bleeding, cerebral infarction, or stroke, Pulmonary Disease includes asthma and chronic obstructive pulmonary disease.

^1^ Factors significant at the p = 0.05 level in two-sided tests were included in the multivariate model

^2^ ADI is calculated in deciles with an ADI of 1 and 10 signifying a neighborhood that is least and most socioeconomically disadvantaged, respectively

^3^ For comorbid conditions the absence of the condition is the referent

**Table 6:** Univariate and multivariate Cox proportional hazards regression analyses of risk factors associated with non-hospital death within 180 days post-hospital discharge (N = 11,406, Number of Non-Hospital Deaths = 261).

			Univariate			Multivariate^1^		
Variable		Non- Hospital Deaths	HR	95% CI	p Value	Adjusted HR	95% CI	p Value
Age Group	18–39 (referent)							
	40–49	20	2.96	1.42, 6.17	0.0039	2.48	1.14, 5.37	0.0215
	50–59	44	4.47	2.31, 8.66	<.0001	3.38	1.69, 6.74	0.0005
	60–69	54	4.78	2.50, 9.14	<.0001	3.02	1.53, 5.96	0.0015
	70–79	47	5.30	2.75, 10.22	<.0001	2.45	1.21, 4.93	0.0125
	80+	85	9.32	4.97, 17.47	<.0001	4.81	2.46, 9.41	<.0001
Sex	Female (referent)							
	Male	139	0.95	0.75, 1.21	0.6902			
Race/Ethnicity	White (referent)							
	Asian American	31	0.68	0.46, 1.01	0.0582			
	Black	24	0.77	0.50, 1.20	0.2485			
	Hispanic	67	0.47	0.35, 0.64	<.0001			
	Other/Unknown	26	0.56	0.37, 0.86	0.0081			
Hospitalization Period	Jan-May 2020	10	1.52	0.63, 3.65	0.3489			
	Jun-Aug 2020	13	0.71	0.31, 1.61	0.4084			
	Sep 2020-Jan 2021	64	1.19	0.61, 2.33	0.6012			
	Feb-May 2021 (referent)							
	Jun-Nov 2021	26	1.57	0.76, 3.26	0.2234			
	Dec 2021-Feb 2022	42	1.45	0.73, 2.88	0.2943			
	Mar-Sep 2022	53	1.83	0.93, 3.60	0.0789			
	Oct 2022-Aug 2023	43	1.26	0.63, 2.51	0.5120			
ADI^**2**^			0.97	0.92, 1.02	0.1876			
**Comorbid Condition** ^**3**^								
Kidney Failure		19	1.18	0.74, 1.88	0.4945			
Major Cardiac Disease		215	3.59	2.61, 4.93	<.0001			
Valvular Cardiac Disease		76	2.54	1.94, 3.31	<.0001			
Cardiac Arrhythmias		157	2.25	1.76, 2.89	<.0001			
Peripheral Vascular Disease		44	1.84	1.33, 2.54	0.0002			
Coagulopathy		120	2.67	2.10, 3.41	<.0001	1.45	1.11, 1.89	0.0065
Anemia		98	2.64	2.05, 3.39	<.0001	1.52	1.16, 1.99	0.0026
Cancer		153	5.81	4.54, 7.44	<.0001	3.93	3.02, 5.11	<.0001
Diabetes		109	1.19	0.93, 1.52	0.1754			
HIV/AIDS		3	0.86	0.28, 2.68	0.7951			
Hypertension		211	2.56	1.88, 3.48	<.0001			
Hyperthyroidism		52	1.38	1.02, 1.86	0.0395			
Chronic Neurological Conditions		81	1.90	1.46, 2.47	<.0001			
Obesity		73	0.97	0.74, 1.27	0.8004			
Paralysis		23	2.07	1.35, 3.17	0.0009			
Chronic Psychoses		4	0.36	0.13, 0.97	0.0435			
Pulmonary Disease		123	1.98	1.56, 2.53	<.0001			
Rheumatoid Arthritis/CVD		28	1.60	1.08, 2.37	0.0190			
Smoking		68	1.39	1.06, 1.84	0.0184			
Solid Organ Transplantation		16	1.30	0.78, 2.16	0.3093			
Liver Disease		87	2.39	1.85, 3.09	<.0001	1.48	1.12, 1.96	0.0064
Cerebrovascular Disease		79	2.43	1.87, 3.16	<.0001	1.53	1.15, 2.03	0.0031
Drug Abuse		21	0.75	0.48, 1.17	0.2044			
**In-Hospital Medication or High Acuity Treatment**								
Remdesivir		126	1.19	0.93, 1.52	0.1601			
Dexamethasone		112	0.84	0.66, 1.07	0.1645			
Oral Blood Thinner		74	2.03	1.55, 2.66	<.0001			
Monoclonal Antibodies		12	1.58	0.88, 2.81	0.1233			
SSRIs		45	1.45	1.05, 2.00	0.0232			
Mechanical Ventilation		34	1.14	0.79, 1.63	0.4787			
Tracheostomy		3	0.77	0.25, 2.41	0.6574			
**In-Hospital Complication**		3						
Deep Vein Thrombosis		2	1.19	0.30, 4.79	0.8049			
Intracerebral Bleeding/Cerebral Infarction/Stroke		45	2.54	1.84, 3.5	<.0001			
Lung Edema		41	2.68	1.92, 3.74	<.0001			
Lung Embolism		33	2.69	1.86, 3.87	<.0001	1.57	1.07, 2.29	0.0211
Acute Myocardial Infarction		75	2.89	2.21, 3.78	<.0001	1.79	1.34, 2.39	<.0001
Septic Shock		141	2.14	1.68, 2.73	<.0001	1.36	1.05, 1.77	0.0207
High Hospital LOS		167	1.87	1.45, 2.40	<.0001	1.38	1.06, 1.80	0.0173
ICU Stay		50	1.12	0.82, 1.52	0.4755			
High ICU LOS		19	1.17	0.73, 1.87	0.5098			

Abbreviations: ADI = area deprivation index, CI = Wald confidence interval, CVD = collagen vascular disease, ECMO = extracorporeal membrane oxygenation, HIV/AIDS = human immunodeficiency virus/acquired immune deficiency syndrome, HR = Wald chi-square hazard ratio, ICU = intensive care unit, LOS = length of stay, SSRIs = selective serotonin reuptake inhibitors, Transplantation = solid organ transplantation, excluding kidney, Stroke = intracerebral bleeding, cerebral infarction, or stroke, Pulmonary Disease includes asthma and chronic obstructive pulmonary disease.

^1^ Factors significant at the p = 0.05 level in two-sided tests were included in the multivariate model

^2^ ADI is calculated in deciles with an ADI of 1 and 10 signifying a neighborhood that is least and most socioeconomically disadvantaged, respectively

^3^ For comorbid conditions the absence of the condition is the referent

**Fig 5:**
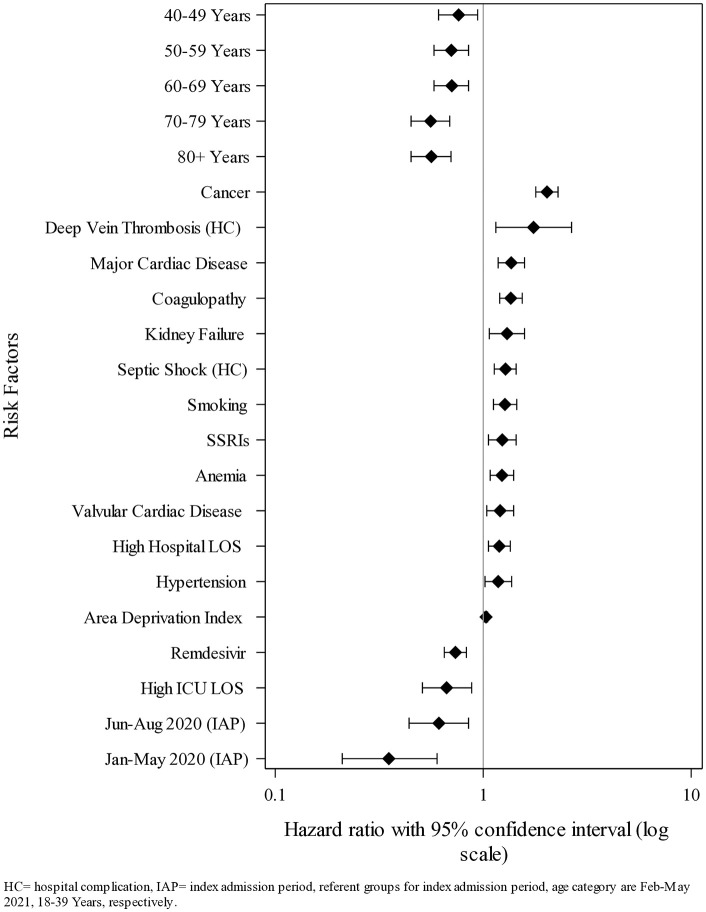
Forest plot showing the multivariate risk factors for hospital readmission within 30 days post-hospital discharge (N = 10,281). HC = hospital complication, IAP = index admission period, referent groups for index admission period, age category are Feb-May 2021, 18-39 years, respectively.

The analysis of risk factors for 30-day readmission post-discharge is shown in Table 7, with multivariate risk factors for this outcome shown in Fig 5. We found cancer to be the factor most associated with a significantly increased risk of 30-day readmission post-discharge (adjusted HR = 2.03, 95% CI 1.79–2.29, p < 0.0001), followed by the hospital complication deep vein thrombosis (adjusted HR = 1.75, 95% CI 1.15–2.66, p = 0.0094), major cardiac disease (adjusted HR = 1.36, 95% CI 1.18–1.58, p < 0.0001), coagulopathy (adjusted HR = 1.36, 95% CI 1.20–1.54, p < 0.0001), kidney failure (adjusted HR = 1.30, 95% CI 1.07–1.58, p= 0.0070), the hospital complication septic shock (adjusted HR=1.28, 95% CI 1.13-1.44, p < 0.0001), being a current or a past smoker (adjusted HR = 1.27, 95% CI 1.12–1.45, p = 0.0003), the administration of SSRIs during the index hospital stay (adjusted HR = 1.23, 95% CI 1.06–1.44, p = 0.0073), anemia (adjusted HR = 1.23, 95% CI 1.08–1.40, p = 0.0021), valvular cardiac disease (adjusted HR = 1.21, 95% CI 1.04–1.40, p = 0.0141), high hospital LOS (adjusted HR = 1.19, 95% CI 1.06–1.35, p = 0.0035), hypertension (adjusted HR = 1.18, 95% CI 1.02–1.37, p = 0.0264), and ADI (adjusted HR = 1.03, 95% CI 1.01–1.05, p = 0.0045). Additionally, we found the following factors to be associated with a significantly reduced risk of 30-day readmission: initial hospitalization during the period Jan-May 2020 (adjusted HR = 0.35, 95% CI 0.21–0.60, p = 0.0001), being 80 years of age or older (adjusted HR = 0.56, 95% CI 0.45–0.70, p < 0.0001), being 70−79 years of age (adjusted HR = 0.56, 95% CI 0.45–0.69, p < 0.0001), initial hospitalization during the period Jun-Aug 2020 (adjusted HR = 0.61, 95% CI 0.44–0.85, p = 0.0029), high ICU length of stay (adjusted HR = 0.67, 95% CI 0.51–0.88, p = 0.0040), being age 50−59 years (adjusted HR = 0.70, 95% CI 0.58–0.85, p = 0.0004), being age 60−69 years (adjusted HR = 0.71, 95% CI 0.58–0.85, p = 0.0003), the administration of Remdesivir during the index hospital stay (adjusted HR = 0.74, 95% CI 0.65–0.83, p < 0.0001), and being age 40−49 years (adjusted HR = 0.76, 95% CI 0.61–0.94, p = 0.0126).

**Table 7:** Univariate and multivariate Cox proportional hazards regression analyses of risk factors associated with hospital readmission within 30 days post-hospital discharge (N = 10,281, Number of Hospital Readmissions = 1,344).

			Univariate			Multivariate^1^		
Variable		Readmissions	HR	95% CI	p Value	Adjusted HR	95% CI	p Value
Age Group	18-39 (referent)							
	40-49	149	0.84	0.69, 1.03	0.0885	0.76	0.61, 0.94	0.0126
	50-59	215	0.83	0.70, 1.00	0.0445	0.70	0.58, 0.85	0.0004
	60-69	284	0.97	0.82, 1.14	0.6901	0.71	0.58, 0.85	0.0003
	70-79	208	0.90	0.75, 1.07	0.2277	0.56	0.45, 0.69	<.0001
	80+	206	0.86	0.72, 1.03	0.0913	0.56	0.45, 0.70	<.0001
Sex	Female (referent)							
	Male	723	0.97	0.87, 1.08	0.6183			
Race/Ethnicity	White (referent)							
	Asian American	162	0.94	0.78, 1.12	0.4898			
	Black	129	1.09	0.90, 1.33	0.3744			
	Hispanic	476	0.88	0.77, 1.01	0.0607			
	Other/Unknown	147	0.84	0.69, 1.01	0.0595			
Hospitalization Period	Jan-May 2020	19	0.39	0.23, 0.64	0.0002	0.35	0.21, 0.60	0.0001
	Jun-Aug 2020	89	0.66	0.49, 0.91	0.0099	0.61	0.44, 0.85	0.0029
	Sep 2020-Jan 2021	301	0.78	0.60, 1.00	0.0542	0.77	0.59, 1.01	0.0623
	Feb-May 2021 (referent)							
	Jun-Nov 2021	100	0.83	0.62, 1.13	0.2425	0.85	0.62, 1.17	0.3284
	Dec 2021-Feb 2022	227	1.09	0.84, 1.42	0.5218	0.93	0.71, 1.23	0.6329
	Mar-Sep 2022	264	1.29	0.99, 1.67	0.0568	1.04	0.79, 1.37	0.7740
	Oct 2022-Aug 2023	272	1.11	0.86, 1.44	0.4186	0.96	0.73, 1.26	0.7457
ADI^**2**^			1.03	1.01, 1.06	0.0016	1.03	1.01, 1.05	0.0045
**Comorbid Condition** ^**3**^								
Kidney Failure		144	1.86	1.57, 2.21	<.0001	1.30	1.07, 1.58	0.0070
Major Cardiac Disease		952	1.89	1.68, 2.13	<.0001	1.36	1.18, 1.58	<.0001
Valvular Cardiac Disease		282	1.66	1.46, 1.90	<.0001	1.21	1.04, 1.40	0.0141
Cardiac Arrhythmias		679	1.54	1.38, 1.71	<.0001			
Peripheral Vascular Disease		190	1.52	1.30, 1.77	<.0001			
Coagulopathy		503	1.93	1.73, 2.16	<.0001	1.36	1.20, 1.54	<.0001
Anemia		382	1.77	1.58, 2.00	<.0001	1.23	1.08, 1.40	0.0021
Cancer		488	2.39	2.14, 2.67	<.0001	2.03	1.79, 2.29	<.0001
Diabetes		536	1.10	0.99, 1.23	0.0889			
HIV/AIDS		20	1.12	0.72, 1.74	0.6232			
Hypertension		922	1.33	1.19, 1.50	<.0001	1.18	1.02, 1.37	0.0264
Hyperthyroidism		246	1.25	1.09, 1.43	0.0017			
Chronic Neurological Conditions		272	1.06	0.93, 1.21	0.3730			
Obesity		401	1.06	0.94, 1.19	0.3206			
Paralysis		74	1.26	0.99, 1.59	0.0562			
Chronic Psychoses		65	1.23	0.96, 1.58	0.1036			
Pulmonary Disease		521	1.43	1.28, 1.59	<.0001			
Rheumatoid Arthritis/CVD		125	1.38	1.15, 1.66	0.0006			
Smoking		354	1.44	1.28, 1.63	<.0001	1.27	1.12, 1.45	0.0003
Solid Organ Transplantation		99	1.63	1.33, 2.00	<.0001			
Liver Disease		344	1.67	1.48, 1.88	<.0001			
Cerebrovascular Disease		255	1.31	1.14, 1.50	<.0001			
Drug Abuse		193	1.47	1.27, 1.72	<.0001			
**In-Hospital Medication or High Acuity Treatment**								
Remdesivir		535	0.83	0.75, 0.93	0.0009	0.74	0.65, 0.83	<.0001
Dexamethasone		567	0.81	0.73, 0.90	0.0001			
Oral Blood Thinner		271	1.30	1.14, 1.49	<.0001			
Monoclonal Antibodies		42	1.04	0.77, 1.42	0.7933			
SSRIs		214	1.34	1.15, 1.55	0.0001	1.23	1.06, 1.44	0.0073
Mechanical Ventilation		157	1.01	0.85, 1.19	0.9095			
ECMO		3	1.10	0.36, 3.41	0.8663			
Tracheostomy		14	0.68	0.40, 1.16	0.1575			
**In-Hospital Complication**		14						
Deep Vein Thrombosis		25	3.33	2.24, 4.94	<.0001	1.75	1.15, 2.66	0.0094
Intracerebral Bleeding/Cerebral Infarction/Stroke		133	1.34	1.12, 1.61	0.0013			
Lung Edema		150	1.85	1.56, 2.19	<.0001			
Lung Embolism		82	1.19	0.95, 1.49	0.1222			
Acute Myocardial Infarction		225	1.45	1.25, 1.67	<.0001			
Septic Shock		602	1.50	1.34, 1.67	<.0001	1.28	1.13, 1.44	<.0001
High Hospital LOS		726	1.24	1.12, 1.38	<.0001	1.19	1.06, 1.35	0.0035
ICU Stay		231	0.98	0.85, 1.13	0.7676			
High ICU LOS		71	0.82	0.65, 1.05	0.1100	0.67	0.51, 0.88	0.0040

Abbreviations: ADI = area deprivation index, CI = Wald confidence interval, CVD = collagen vascular disease, ECMO = extracorporeal membrane oxygenation, HIV/AIDS = human immunodeficiency virus/acquired immune deficiency syndrome, HR = Wald chi-square hazard ratio, ICU = intensive care unit, LOS = length of stay, SSRIs = selective serotonin reuptake inhibitors, Transplantation = solid organ transplantation, excluding kidney, Stroke = intracerebral bleeding, cerebral infarction, or stroke, Pulmonary Disease includes asthma and chronic obstructive pulmonary disease.

^1^ Factors significant at the p = 0.05 level in two-sided tests were included in the multivariate model

^2^ ADI is calculated in deciles with an ADI of 1 and 10 signifying a neighborhood that is least and most socioeconomically disadvantaged, respectively

^3^ For comorbid conditions the absence of the condition is the referent

## Discussion

Using a large database that spans the five academic medical centers of the University of California, we sought to determine if ESRD was independently associated with a higher risk for hospital, non-hospital, and overall death within 180 days post-discharge, and 30-day hospital readmission for those diagnosed with COVID-19. Individuals receiving hemodialysis for ESRD are known to have a high 30-day readmission rate, with the reason for readmission not necessarily related to the reason for the index hospitalization [13]. A similar pattern exists for COVID-19 related hospitalizations. Our retrospective study spanned almost the entire period of the COVID-19 pandemic. As a result, the dominant strain of SARS-CoV-2 in the community, and that which caused hospitalizations during the pandemic, likely changed over the course of the study. However, including a factor for index hospitalization period did not substantially affect our results. Individuals with ESRD have many of the comorbid conditions linked with worse outcomes from COVID-19 and are more frequently readmitted post-hospitalization. It is not unexpected therefore, that as was found by Kingery et al and Verna et al who studied patients with ESRD, and Akbari et al who studied patients with kidney disease, we found a diagnosis of ESRD, upon adjusting for demographic variables, comorbid conditions, and other covariates, to be independently associated with a significantly higher risk of 30-day readmission for individuals hospitalized with COVID-19. The association which we found (adjusted HR 1.36, 95% CI 1.14–1.63, p = 0.001) however, was lower in magnitude than that found by Kingery et al (adjusted HR 2.94, 95% CI 1.78–4.84, p < 0.0001), Verna et al (adjusted OR 2.27, 95% CI 1.81–2.86), and Akbari et al (adjusted OR 2.52, 95% CI 1.23–2.85, p < 0.0001). The magnitude of the association between 30-day readmission and presence of ESRD which we found was also nearly identical to the association found by Huang et al between 30-day readmission and presence of renal disease (N = 1,280, adjusted OR 1.35, 95% CI 0.81–2.26, p = 0.25).

Our second finding was that the presence of ESRD was independently associated with a higher risk of in-hospital and overall death within 180 days (adjusted HR 1.36, 95% CI 0.78–2.37, and adjusted HR 1.05, 95% CI 0.73–1.51, respectively) although we did not find these risks to be statistically significant. This may be possibly due to the immune system compromise of individuals with ESRD, and the resulting reduction of cytokine storms and systemic inflammation [14]. Our finding for the risk of overall death within 180 days post-discharge was also lower than that found by Kingery et al for the risk of overall death within 30 days (adjusted HR 1.22, 95% CI 0.29–5.12, p = 0.789), and Verna et al for the risk of death during hospital readmission (adjusted OR 1.43, 95% CI 0.63–3.24), perhaps because our study spanned the period January 2020 through August 2023, and the other two studies were conducted early in year 2020, when a higher mortality rate from COVID-19 was observed.

Our third finding was that the presence of ESRD was independently associated with a lower risk of non-hospital death within 180 days (adjusted HR 0.89, 95% CI 0.55–1.44 for non-hospital death), although again, we did not find this risk to be statistically significant.

When we examined the associations between our risk factors and our study outcomes, we found that the comorbid conditions cancer and anemia, high hospital length of stay, and the hospital complications septic shock and acute myocardial infarction were significantly associated with increased hazard of both hospital and non-hospital death within 180 days post-discharge. Furthermore, each of the first four of these risk factors were also significantly associated with an increased hazard of 30-day readmission post-discharge. Interestingly, we found that the comorbid conditions valvular cardiac disease and hypertension, the hospital complication deep vein thrombosis, smoking, in-hospital use of SSRIs, and higher ADI were factors significantly associated with an increased hazard of 30-day hospital readmission, but that these factors were not significantly associated with an increased hazard of either hospital or non-hospital death within 180 days. Similarly, high ICU length of stay, in-hospital use of Remdesivir, and index hospitalization in the period January-August 2020 were factors significantly associated with a reduced hazard of 30-day hospital readmission, but not with a significantly reduced hazard of either hospital or non-hospital death within 180 days. We found that being 40 or more years of age was significantly associated with a reduced hazard of 30-day hospital readmission, but in contrast, was also significantly associated with an increased hazard of non-hospital death within 180 days.

The strengths of this study include its large cohort size, its diverse racial groups, 3.5-year long duration, and the geographical and population range covered by the 5 academic medical centers in California from which we enrolled patients. We also included many comorbid conditions and complications, and we were able to capture key therapeutics used to treat COVID-19 patients during the period covered by our study. Like all studies of this kind, the retrospective nature of our study is a limitation. Additional limitations include that comorbidity ascertainment was performed using diagnostic codes, which vary in their degree of accuracy and completeness, and which we were not able to validate with chart review due to the pseudonymized nature of the dataset. Given the nature of the UC CORDS database, it was also not possible to study some patient-level variables, including blood type, familial history, and genetic factors, each of which might have contributed to residual confounding for the associations which we studied. Additionally, since this study spans 3.5 years of the pandemic, patient care management with increased experience with COVID-19 care may have changed clinical practices over time [15], possibly resulting in aggregation bias. Also, some patients with COVID-19 admitted to one of the University of California medical centers during our study period may have had false negative or no recorded COVID-19 test results, possibly resulting in selection bias. The wider availability and use of COVID-19 vaccines over the study period could also conceivably have contributed to aggregation bias. Vaccination against SARS-CoV-2, which reduces severe disease, hospitalizations, and death, was introduced in California in late December 2020. We sought to identify hospitalized patients who had received 1 or more vaccinations and only found 2,265 such patients (19.9%). Our database identified patients who had a vaccination recorded within (or information transferred to) the UC health system, whereas population vaccination was primarily through community vaccination clinics, pharmacy chains, and dialysis clinics. Therefore, our database likely undercounted the number of vaccinated patients. It is also possible that the vaccines worked effectively, reducing hospitalizations for COVID-19 [16]. Nonetheless, adjusting for limited information about COVID-19 vaccination status in the analysis did not affect our results.

In conclusion, we found that after adjusting for demographic factors, hospitalization period, patient comorbidities, and other covariates, ESRD status was not found to significantly increase the risk for hospital, non-hospital, or overall death within 180 days post-discharge (adjusted hazard ratio (HR) 1.36, 95% CI 0.78–2.37, p = 0.278; adjusted HR 0.89, 95% CI 0.55–1.44, p = 0.634; and adjusted HR 1.05, 95% CI 0.73–1.51, p = 0.795, respectively), but was found to significantly increase the risk of 30-day hospital readmission (adjusted HR 1.36, 95% CI 1.14–1.63, p = 0.001). We recommend that caution be exercised when discharging ESRD patients, especially those with a history of major cardiac disease, anemia, or coagulopathy. We also suggest that the etiology of long-haul COVID-19 and of ESRD patients post hospitalization for COVID-19 be further studied to ascertain what extent long-haul COVID-19 impacts clinical outcomes for these patients.

## Abbreviations

ADI: area deprivation index
CI: confidence interval
COVID-19: coronavirus disease 2019
ECMO: extracorporeal membrane oxygenation
ESRD: end stage renal disease
HR: hazard ratio
ICD-10-CM: International Classification of Diseases, 10th Revision, Clinical Modification
ICU: intensive care unit
LOS: length of stay
SARS-CoV-2: Severe Acute Respiratory Syndrome Coronavirus 2
SSRIs: selective serotonin reuptake inhibitors
UC: University of California.
